# S03-2 The Child-COOP Denmark study: using physical literacy to guide and evaluate a systemic approach to health promotion

**DOI:** 10.1093/eurpub/ckac093.013

**Published:** 2022-08-29

**Authors:** Knud Ryom, Helene Kirkegaard, Jane Nautrup Østergaard, Helle Terkildsen Maindal

**Affiliations:** 1 Department of Public Health, Applied Public Health Research, Aarhus University, Aarhus, Denmark; 2 Steno Diabetes Center Copenhagen, Copenhagen, Denmark

**Keywords:** Intervention, physical activity, physical literacy, systemic approach

## Abstract

**Background:**

Children’s health is generally considered a complex interplay between multiple factors. Interventions building on community-based participatory research and system dynamics have shown promising potential in improving children's health behavior and well-being. This presentation aims to present how physical literacy can be used to guide and evaluate a systemic approach to health promotion.

**Methods:**

System dynamics techniques such as group model building, is used to engage a whole community in a rural area of Denmark and develop local actions for enhancing among other physical activity. A central health outcome in The Child-COOP Denmark study is physical literacy, which is measured by using DAPL (the Danish version of CAPL). Physical literacy will be used as a central element to guide and evaluate the project. The evaluation design includes repeated measures of childhood health behavior, physical literacy and well-being among 100 children (6-13 y) attending the local primary school. With data collection at baseline and at 2 and 4 years of follow up.

**Results:**

Furthermore, physical literacy results throughout the project period will also be used to guide new local actions in the environment aiming to enhance the local children’s health, well-being and physical activity.

**Discussion:**

The potential of using physical literacy measures to guide and evaluate a participatory systems approach in order to solve complex health problems, is discussed and debated with the audience.

